# Polysubstance mortality trends in White and Black Americans during the opioid epidemic, 1999–2018

**DOI:** 10.1186/s12889-023-17563-x

**Published:** 2024-01-07

**Authors:** Marwa Rawy, Gergis Abdalla, Kevin Look

**Affiliations:** 1 University of Wisconsin–Madison, Madison, USA; 2 Claude Bernard University Lyon 1, Lyon, France

**Keywords:** Polysubstance mortality, Joinpoint trend analysis, Opioids, Stimulants, Benzodiazepines, Racial disparities

## Abstract

**Background:**

Psychoactive drug combinations are increasingly contributing to overdose deaths among White and Black Americans. To understand the evolving nature of overdose crisis, inform policies, and develop tailored and equitable interventions, this study provides a comprehensive assessment of polysubstance mortality trends by race and sex during the opioid epidemic.

**Methods:**

We used serial cross-sectional US mortality data for White and Black populations from 1999 through 2018 to calculate annual age-adjusted death rates (AADR) involving any opioid, opioid subtypes, benzodiazepines, cocaine, psychostimulants, or combinations of these drugs, stratified by race and sex. Trend changes in AADR were analyzed using joinpoint regression models and expressed as average annual percent change (AAPC) during each period of the three waves of the opioid epidemic: 1999–2010 (wave 1), 2010–2013 (wave 2), and 2013–2018 (wave 3). Prevalence measures assessed the percent co-involvement of an investigated drug in the overall death from another drug.

**Results:**

Polysubstance mortality has shifted from a modest rise in death rates due to benzodiazepine-opioid overdoses among White persons (wave 1) to a substantial increase in death rates due to illicit drug combinations impacting both White and Black populations (wave 3). Concurrent cocaine-opioid use had the highest polysubstance mortality rates in 2018 among Black (5.28 per 100,000) and White (3.53 per 100,000) persons. The steepest increase in death rates during wave 3 was observed across all psychoactive drugs when combined with synthetic opioids in both racial groups. Since 2013, Black persons have died faster from cocaine-opioid and psychostimulant-opioid overdoses. Between 2013 and 2018, opioids were highly prevalent in cocaine-related deaths, increasing by 33% in White persons compared to 135% in Blacks. By 2018, opioids contributed to approximately half of psychostimulant and 85% of benzodiazepine fatal overdoses in both groups. The magnitude and type of drug combinations with the highest death rates differed by race and sex, with Black men exhibiting the highest overdose burden beginning in 2013.

**Conclusions:**

The current drug crisis should be considered in the context of polysubstance use. Effective measures and policies are needed to curb synthetic opioid-involved deaths and address disparate mortality rates in Black communities.

**Supplementary Information:**

The online version contains supplementary material available at 10.1186/s12889-023-17563-x.

## Background

Drug overdose has claimed the lives of almost a million Americans since the beginning of the opioid epidemic in 1999 and continues to escalate as a public health emergency [1]. Recent data estimated an increase in drug overdose deaths from 67,367 in 2018 to more than 107,000 in 2021 [2]. During this period, opioids were involved in approximately 75% of overdose deaths [3, 4]. The Centers for Disease Control and Prevention (CDC) characterized mortality trends involving opioids into three waves based on the opioid subtype driving overdose death. The first wave spanned from 1999 to 2010 and was primarily driven by prescription opioid overdose. Deaths due to heroin triggered a second wave in 2010, and a third wave began in 2013 with a surge in deaths due to synthetic opioids, mainly illicitly manufactured fentanyl and its analogs [4]. Public health surveillance reports and studies on drug use and mortality have documented the emerging threat from stimulants and polysubstance use in what some experts termed as the “fourth wave” of the opioid crisis [5–7].

Polysubstance use is a risk factor for drug overdose [8]. It refers to the exposure to more than one drug, together or within a short time period, with or without the person’s knowledge, for either therapeutic or recreational purposes [9]. It might involve the concurrent use of multiple opioids or the combination of an opioid and a nonopioid drug. In a national survey of 15,741 patients entering treatment for opioid use disorder (OUD), almost all participants reported concurrently using at least one nonopioid drug in the previous month [10]. The high potency, low cost, and ease of production of the synthetic opioid fentanyl facilitated mixing it with other drugs and contributed to recent spikes in overdose deaths [11–13].

Polysubstance use has complicated the opioid epidemic by challenging mitigating policies and prevention strategies and adversely affecting patient outcomes in opioid treatment programs [14]. The concurrent use of various substances in patients with OUD has been associated with a lower likelihood of receiving medications for OUD and reduced treatment retention [14–16]. Unlike the effective medication-assisted therapy for OUD, there are currently no approved or effective pharmacologic treatments for addressing other substance use disorders or polysubstance use [17, 18]. Harm reduction strategies like naloxone only reverse overdoses from an opioid and may not be effective in conventional doses in the presence of the lethal, rapidly acting fentanyl. Furthermore, opioid-naïve drug users are not a typical target for naloxone distribution and are more likely to overdose when inadvertently consuming fentanyl-laced drugs [19].

The impact of the opioid crisis on White and Black Americans has varied over time. The dramatic increase in opioid-related deaths during wave 1 resulted from misleading pharmaceutical marketing and opioid analgesics overprescribing that disproportionately affected White persons, especially in suburban and rural areas. This stereotyped the opioid crisis as a “White epidemic” and marginalized Black persons from the opioid narrative presuming they were protected due to the inherent disparities in prescribing narcotic analgesics to Black patients [20–22]. Both racial groups, however, experienced unprecedented death rates due to heroin and synthetic opioids during the second and third waves, respectively [23]. Only recently has the opioid crisis in Black communities gained attention with the marked increase in opioid death rates among Black persons over the past decade [22, 24, 25]. Furr-Holden and colleagues investigated all opioid-involved death rates by race between 1999 and 2018. Unlike the increasing trends in White persons that aligned with the three-wave depiction of the opioid epidemic, death rates among Black persons were low and flat and then accelerated sharply starting in 2012 at a higher rate than their White counterparts [26]. A recent study by Kariisa and colleagues attributed the sharp increase in death rates from cocaine overdose since 2013 in both White and Black persons to opioid co-involvement, with Black individuals experiencing disproportionally higher rates of cocaine- and opioid-involved deaths [27].

The growing evidence of polysubstance use as a cause of overdose death necessitates a comprehensive assessment to describe and quantify combinations driving overdose mortality among subgroups of the US population. Epidemiological studies and reports investigating overdose deaths have primarily focused on opioids with limited emphasis on polysubstance overdose. Racial differences in polysubstance mortality and the interaction between race and sex have also not been adequately described despite the surge in overdose death rates among Black Americans. This inadequate characterization of overdose mortality poses risks to the success of policies and public health measures targeting addiction and drug crisis. Additionally, there remains a knowledge gap in how polysubstance mortality patterns have evolved over time, particularly when mapped to the three distinct waves that define the opioid epidemic. Strengthening our understanding of the epidemic is one of the five major priorities the US Department of Health and Human Services has set to respond to the opioid crisis [28]. Thus, this study aims to investigate and compare the changes in polysubstance mortality among White and Black Americans due to different drug combinations as compared to the overall mortality from psychoactive drugs within the context of the three waves of the opioid epidemic. The comparison of trends from the two perspectives of polysubstance and overall mortality is important because reporting the latter has been the standard practice in overdose mortality research. As a secondary objective, we assess trends in polysubstance mortality at the intersection of race and sex. This multifaceted approach provides a comprehensive characterization and understanding of polysubstance mortality patterns among diverse subpopulations. It serves as a preliminary step towards identifying at-risk groups and developing targeted treatment and harm reduction measures to effectively address the complex landscape of drug crisis.

## Methods

This population-based cross-sectional study examined mortality trends in White and Black Americans that involved combinations of psychoactive therapeutic and/or illicit drugs commonly reported as a cause of overdose deaths [29]. Drug classes analyzed were opioids, benzodiazepines, cocaine, and psychostimulants with abuse potential other than cocaine (mainly including methamphetamine, hereafter referred to as psychostimulants). In addition to identifying deaths from combinations co-involving any opioid drug, three opioid subtypes (prescription opioids, heroin, and synthetic opioids) were investigated given their role in fueling the opioid epidemic. This study followed the Strengthening the Reporting of Observational Studies in Epidemiology (STROBE) guidelines for reporting cross-sectional studies and the National Center for Health Statistics (NCHS) guidelines for analysis of trends [30, 31]. It was exempt from review by the institutional review board at the University of Wisconsin-Madison as it used publicly available de-identified data.

### Data source and study population

We used 1999 through 2018 data from the National Vital Statistics System (NVSS) multiple cause-of-death files, population-level microdata compiled by the NCHS from death certificates filed in all 50 US states and the District of Columbia [32]. The NVSS mortality data provide the most complete data on deaths in the United States and have been extensively used in surveillance reports and studies investigating drug overdose deaths [3, 5, 12, 33]. Each observation includes the demographic information of the deceased US resident, a single underlying cause that directly led to death, and up to 20 multiple causes contributing to death. Underlying causes of death due to drug poisoning or overdose were identified based on the International Classification of Diseases, 10th revision (ICD-10) codes X40–X44 (unintentional), X60–X64 (suicide), X85 (homicide), or Y10–Y14 (undetermined intent). Overdose deaths were then restricted to the specific drugs investigated in this study using the corresponding ICD-10 multiple-cause-of-death codes listed in Additional File Table 1. We defined “polysubstance death” as a drug-related death record that included codes from at least two investigated drugs. “Overall mortality” from a drug refers to all deaths involving this specific drug, whether alone or in combination with any other drug.

### Statistical analysis

Data were analyzed using R for Windows (version 3.6.0) and the Joinpoint Regression Program (version 4.8.0.1) [34]. First, data were aggregated to identify annual death counts for Black and White populations stratified by 5-year age groups (i.e., < 5, 5–9, …, 85 +) for each investigated drug overall and for combinations involving at least two drugs. Using the US Census Bureau bridged-race national population estimates as the denominator [35], we computed the annual death rates per 100,000 population that were age-adjusted to the 2000 US standard population using the direct method [36].

Joinpoint regression analysis was used to fit weighted least square regression models on a log-linear scale to the observed annual age-adjusted death rate (AADR) stratified by race (White and Black persons), sex (men, women, all persons), and specific drug/drug combination categories. Allowing a maximum of three joinpoints and using a Monte Carlo Permutation test, we identified the parsimonious number of joinpoints that mark the breaking points for years when mortality trends significantly changed during the study period. The log-linear model facilitated trend comparison by estimating the Annual Percent Change (APC) and associated 95% confidence interval from the slope of each fitted trend segment, which quantifies a constant change in death rate over time for each segment. The Average Annual Percent Change (AAPC), a weighted average of APCs, provided a summary of the APCs over three pre-specified fixed intervals (1999–2010, 2010–2013, and 2013–2018), which correspond to the CDC’s defined three waves of the opioid epidemic. The AAPC difference between Black and White persons for each of these three intervals represents disparities in trends. The program uses t-tests to assess the statistical significance of APCs, AAPCs, and Black-White differences in AAPCs. All analyses were carried out using a two-sided statistical significance level of 0.05. A trend segment was described as increasing/rising or decreasing/declining if the *p*-value of the slope was statistically significant.

Prevalence measures identified the percent co-involvement of an investigated drug in the overall death of another for all White and Black persons and for men and women of each racial group. For example, the prevalence of opioids in cocaine overdose deaths was calculated in White and Black populations separately by dividing the number of deaths in a year in which at least one opioid drug and cocaine were concurrently reported as a cause of death by the overall death counts that involved cocaine for that year.

## Results

### AADR and mortality trends co-involving any opioid

**Fig. 1:**
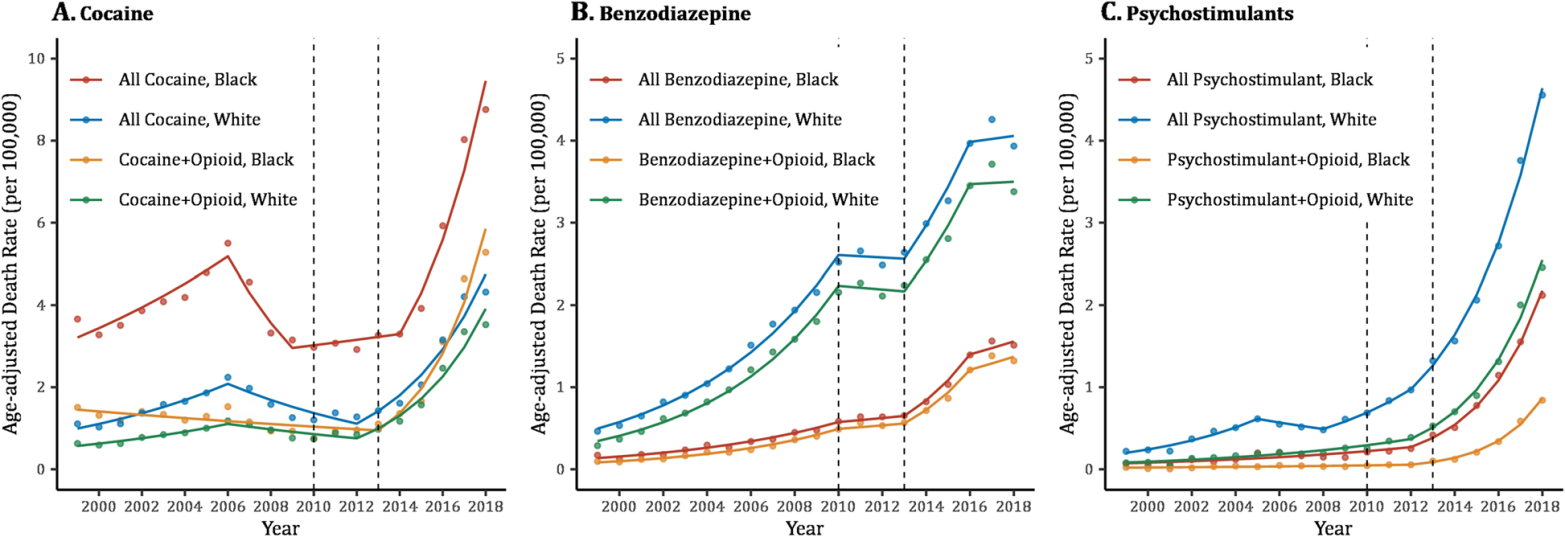
Mortality trends from (**A**) cocaine, (**B**) benzodiazepines, and (**C**) psychostimulants overall and in combination with opioids by race, 1999–2018. Notes: Dots represent observed age-adjusted death rates, while solid lines represent modeled rates. Trend segments were fitted using joinpoint regression modeling. Segments are connected at joinpoints denoting years of significant changes in trend. The slope of each segment represents the Annual Percent Change (APC). Vertical dashed lines at 2010 and 2013 mark the beginning of the second and the third waves of the opioid epidemic, respectively. Death rates were calculated per 100,000 population and adjusted to the 2000 US standard population. Overall mortality from a specific drug is all deaths involving this drug whether alone or when combined with any other drug of abuse. Data are from the Centers for Disease Control and Prevention [32, 35]

Figure 1 and Table 1 display mortality trends in Black and White persons due to three nonopioid classes (cocaine, benzodiazepines, and psychostimulants) overall and when combined with at least one opioid drug. Of the investigated nonopioids, cocaine-related overdose resulted in the highest AADR per 100,000 in 2018 in Black persons (8.76), which doubled that of Whites (4.31). Conversely, psychostimulants and benzodiazepines had more than two-fold higher death rates among White persons (4.56 and 3.93, respectively) compared to Blacks (2.12 and 1.51, respectively) in 2018. When deaths were limited only to records concurrently involving an opioid, both Black and White persons experienced the highest death rates from cocaine-opioid combinations in 2018 (5.28 vs 3.53, respectively).

**Table 1:** Mortality trends from cocaine, benzodiazepines, and psychostimulants overall and in combination with opioids by race, 1999–2018

Cause of drug-related death	Time interval^a^	White				Black / African American				AAPC Black-White diff	*p*-value
		Opioid prevalence (%)^b^	Age-adjusted death rate per 100,000 population^c^		AAPC (95% CI)	Opioid prevalence (%)^b^	Age-adjusted death rate per 100,000 population^c^		AAPC (95%CI)		
			Start	End			Start	End			
All Cocaine	1999–2010	60.42	1.11	1.20	2.97 (-1.18, 7.29)	25.24	3.66	2.97	-0.55 (-7.51, 6.92)	-3.52	0.41
	2010–2013	67.05	1.20	1.43	1.09 (-4.06, 6.52)	33.40	2.97	3.27	2.20 (-7.39, 12.80)	1.11	0.83
	2013–2018	80.29	1.43	4.31	27.37 (21.02, 34.05)^d^	59.29	3.27	8.76	24.00 (17.81, 30.51)^d^	-3.37	0.44
Cocaine + Opioid	1999–2010		0.63	0.74	3.78 (-0.79, 8.57)		1.51	0.75	-3.01 ( -5.37, -0.59)^d^	-6.79^d^	0.01
	2010–2013		0.74	0.98	5.02 (-0.67, 11.05)		0.75	1.10	-3.01 (-5.37, -0.59)^d^	-8.03^d^	0.01
	2013–2018		0.98	3.53	31.56 (25.23, 38.20)^d^		1.10	5.28	43.93 (33.89, 54.71)^d^	12.37^d^	0.03
All Benzodiazepine	1999–2010	84.97	0.46	2.52	16.30 (15.03, 17.59)^d^	84.36	0.17	0.58	13.99 (11.47, 16.56)^d^	-2.32	0.07
	2010–2013	84.19	2.52	2.64	-0.58 (-10.15, 10.01)	86.57	0.58	0.65	4.15 (-14.52, 26.91)	4.73	0.64
	2013–2018	85.12	2.64	3.93	9.64 (3.76, 15.84)^d^	87.97	0.65	1.51	19.09 (8.38, 30.86)^d^	9.45	0.15
Benzodiazepine + Opioid	1999–2010		0.29	2.16	18.55 (16.79, 20.34)^d^		0.10	0.49	17.47 (15.65, 19.31)^d^	-1.08	0.34
	2010–2013		2.16	2.24	-1.01 (-13.00, 12.63)		0.49	0.57	4.28 (-8.03, 18.24)	5.29	0.51
	2013–2018		2.24	3.38	10.05 (2.62, 18.03)^d^		0.57	1.32	19.71 (12.75, 27.09)^d^	9.65	0.07
All psychostimulant	1999–2010	36.30	0.22	0.69	11.92 (3.29, 21.26)^d^	22.09	0.07	0.21	10.58 (5.85, 15.52)^d^	-1.34	0.79
	2010–2013	38.86	0.69	1.32	22.28 (13.41, 31.85)^d^	25.14	0.21	0.42	20.08 (16.48, 23.79)^d^	-2.20	0.66
	2013–2018	52.29	1.32	4.56	29.72 (26.63, 32.88)^d^	40.19	0.42	2.12	41.61 (35.09, 48.43)^d^	11.89^d^	0.00
Psychostimulant + Opioid	1999–2010		0.08	0.25	12.21 (9.32, 15.17)^d^		0.02	0.04	8.14 (2.53, 14.05)^d^	-4.07	0.18
	2010–2013		0.25	0.53	20.21 (17.99, 22.47)^d^		0.04	0.10	22.68 (18.30, 27.22)^d^	2.47	0.33
	2013–2018		0.53	2.45	37.97 (33.68, 42.39)^d^		0.10	0.84	57.87 (49.84, 66.33)^d^	19.90^d^	0.00

Data source: Centers for Disease Control and Prevention [32, 35]

Abbreviation: *AAPC* Average annual percent change; *AAPC Black-White diff* Average annual percent change difference between Black and White populations

Note: Overall mortality from a specific drug is all deaths involving this drug whether alone or when combined with any other drug

^a^Time intervals represent the CDC definition of the three waves of the current opioid epidemic: wave 1 (1999-2010), wave 2 (2010-2013), wave 3 (2013-2018)

^b^Percent prevalence for a single year at the end of the time intervals (i.e., 2010, 2013, 2018)

^c^Rates are for single years at time interval boundaries. Rates were adjusted to the 2000 US standard population

^d^AAPC is statistically significant from zero (2-sided *P* < 0.05). Mortality trends were evaluated using the Joinpoint Regression Program (Version 4.8.0.1)

Overall cocaine death rates were consistently higher in Black than White persons throughout the study period and markedly increased during the third wave of the opioid epidemic at a similar pace in both racial groups (AAPC Black-White difference = -3.37, *p* = 0.44) (Fig. 1, Table 1). However, Black mortality rose at a faster rate from concurrent cocaine-opioid overdoses during wave 3 (AAPC Black-White difference = 12.37, *p* = 0.03). Unlike cocaine, death rates from benzodiazepines or psychostimulants overdose were consistently higher in White than Black persons during the entire study period (Fig. 1). AADRs from benzodiazepine-opioid combinations were very close to the overall rates from benzodiazepines and their trends showed similar patterns of change including two periods of significant increases during waves 1 and 3, with no significant racial differences. Psychostimulant mortality trended upwards during all three waves, with an accelerated increase during wave 3 that was more pronounced among Black persons (AAPC Black-White difference = 11.89, *p* < 0.001) (Table 1). Psychostimulant-opioid combinations revealed widening racial disparities in mortality trends during wave 3 (AAPC Black-White difference = 19.90, *p* < 0.001).

### AADR and mortality trends co-involving specific opioid subtypes

**Fig. 2:**
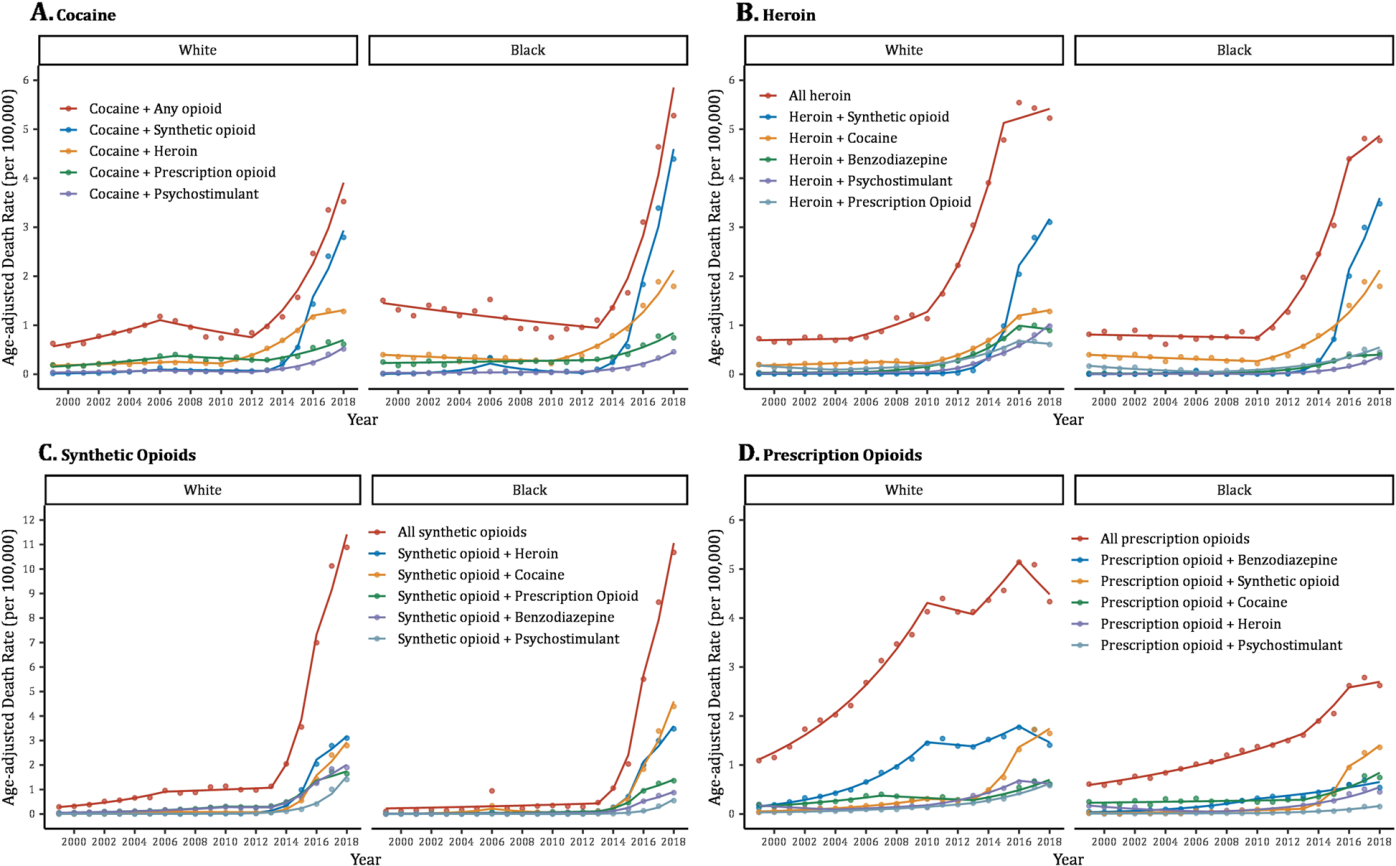
Mortality trends due to combinations of opioids and other psychoactive drugs by opioid subtype and race, 1999–2018. Notes: Dots represent observed age-adjusted death rates, while solid lines represent modeled rates. Trend segments were fitted using joinpoint regression modeling. Segments are connected at joinpoints denoting years of significant changes in trend. Death rates were calculated per 100,000 population and adjusted to the 2000 US standard population. Opioid subtypes are heroin, prescription opioids, and synthetic opioids. Data are from the Centers for Disease Control and Prevention [32, 35]

Synthetic opioids had the highest AADR among all investigated opioid/nonopioid drugs (Fig. 2, Table 2) exceeding 10 per 100,000 in both racial groups in 2018, up from 1.13 (White persons) and 0.47 (Black persons) in 2013. This steep increase was an average of 60.43% per year in White persons compared to 90.85% in Black counterparts; the AAPC Black-White difference was statistically significant in a sensitivity analysis excluding an outlier data point in 2006 (Additional File Table 2). Synthetic opioids substantially influenced polysubstance mortality in both races, especially when combined with cocaine or heroin. The AADR due to synthetic opioid-cocaine combinations increased from 0.08 to 2.79 in White persons and from 0.10 to 4.39 in Black persons between 2013 and 2018 at average rates of 111.82% and 123.45% per year, respectively (AAPC Black-White difference = 11.63, *p* = 0.42) (Table 2). As for synthetic opioid-heroin combinations, the AADR increased from 0.08 to 3.10 in White persons compared to 0.06 to 3.48 in Black persons at average rates of 88.42% and 114.52% per year, respectively (AAPC Black-White difference = 26.11, *p* = 0.09). Even though AADR in 2018 from synthetic opioid-psychostimulant overdose in White (1.41) and Black (0.55) persons were not as high as other combinations involving synthetic opioids, rates had sharply increased since 2013 with Black persons experiencing a disproportionately faster average annual increase (122.75%) compared to Whites (87.42%) (AAPC Black-White difference = 35.33, *p* < 0.001) (Table 2).

**Table 2:** Mortality trends due to combinations of opioids and other psychoactive drugs by opioid subtype and race, 1999–2018

Drug/drug combination	White					Black/African American					AAPC Black-White diff	*p*-value
	Age-adjusted death rate^a^				AAPC (95%CI) (2013–2018)	Age-adjusted death rate^a^				AAPC (95%CI) (2013–2018)		
	1999	2010	2013	2018		1999	2010	2013	2018			
**Cocaine**												
Overall	1.11	1.20	1.43	4.32	27.37 (21.02, 34.05)^b^	3.66	2.97	3.27	8.76	24.00 (17.81, 30.51)^b^	-3.37	0.44
+ Any opioid	0.63	0.74	0.98	3.53	31.56 (25.23, 38.20)^b^	1.51	0.75	1.10	5.28	43.93 (33.89, 54.71)^b^	12.37^b^	0.03
+ Heroin	0.19	0.20	0.53	1.28	20.48 (16.43, 24.66)^b^	0.40	0.22	0.57	1.79	29.54 (24.73, 34.54)^b^	9.06^b^	0.00
+ Synthetic opioids	0.02	0.06	0.08	2.79	111.82 (94.62, 130.54)^b^	0.01	0.04	0.10	4.39	123.45 (102.90, 146.08)^b^	11.63	0.42
+ Prescription opioids	0.19	0.31	0.29	0.61	19.66 (11.12, 28.87)^b^	0.25	0.24	0.31	0.75	23.63 (14.72, 33.23)^b^	3.96	0.51
+ Psychostimulants	0.04	0.04	0.08	0.52	48.81 (40.12, 58.04)^b^	0.02	0.04	0.08	0.46	44.29 (33.22, 56.27)^b^	-4.52	0.51
**Heroin**												
Overall	0.73	1.13	3.04	5.23	13.01 (10.37, 15.72)^b^	0.82	0.74	1.98	4.77	21.93 (15.55, 28.65)^b^	8.91^b^	0.01
+ Synthetic opioids	0.01	0.02	0.08	3.10	88.42 (67.11, 112.44)^b^	0.00	0.00	0.06	3.48	114.52 (95.11, 135.86)^b^	26.11	0.09
+ Prescription opioids	0.20	0.15	0.38	0.61	12.16 (5.15, 19.64)^b^	0.17	0.06	0.18	0.45	25.05 (20.10, 30.21)^b^	12.89^b^	0.00
+ Benzodiazepines	0.03	0.12	0.42	0.89	18.35 (11.79, 25.29)^b^	0.02	0.05	0.11	0.40	24.90 (18.69, 31.43)^b^	6.55	0.17
+ Cocaine	0.19	0.20	0.53	1.28	20.48 (16.43, 24.66)^b^	0.40	0.22	0.57	1.79	29.54 (24.73, 34.54)^b^	9.06^b^	0.00
+ Psychostimulants	0.02	0.04	0.21	0.98	37.88 (33.84, 42.03)^b^	0.01	0.01	0.04	0.34	54.70 (47.41, 62.36)^b^	16.83^b^	0.00
**Synthetic opioids**												
Overall	0.29	1.14	1.13	10.89	60.43 (45.99, 76.28)^b^	0.11	0.35	0.47	10.68	90.85 (16.53, 212.58)^b^	30.43	0.53
+ Heroin	0.01	0.02	0.08	3.10	88.42 (67.11, 112.44)^b^	0.00	0.00	0.06	3.48	114.52 (95.11, 135.86)^b^	26.11	0.09
+ Prescription opioids	0.05	0.31	0.33	1.65	43.06 (34.18, 52.52)^b^	0.02	0.09	0.13	1.36	66.45 (38.34, 100.27)^b^	23.39	0.15
+ Benzodiazepines	0.05	0.29	0.30	1.90	49.37 (39.99, 59.38)^b^	0.02	0.05	0.08	0.87	72.71 (50.74, 97.89)^b^	23.34	0.07
+ Cocaine	0.02	0.06	0.08	2.79	111.82 (94.62, 130.54)^b^	0.01	0.04	0.10	4.39	123.45 (102.90, 146.08)^b^	11.63	0.42
+ Psychostimulants	0.00	0.03	0.06	1.41	87.42 (74.89, 100.85)^b^	0.00	0.00	0.01	0.55	122.75 (101.60, 146.13)^b^	35.33^b^	0.00
**Prescription opioids**												
Overall	1.09	4.13	4.13	4.34	1.92 (-4.81, 9.12)	0.61	1.38	1.61	2.62	10.44 (4.60, 16.61)^b^	8.52	0.07
+ Synthetic opioids	0.05	0.31	0.33	1.65	43.06 (34.18, 52.52)^b^	0.02	0.09	0.13	1.36	66.45 (38.34, 100.27)^b^	23.39	0.15
+ Heroin	0.20	0.15	0.38	0.61	12.16 (5.15, 19.64)^b^	0.17	0.06	0.18	0.45	25.05 (20.10, 30.21)^b^	12.89^b^	0.00
+ Benzodiazepines	0.13	1.45	1.37	1.41	1.15 (-6.30, 9.20)	0.05	0.32	0.36	0.54	9.67 (6.26, 13.20)^b^	8.52^b^	0.05
+ Cocaine	0.19	0.31	0.29	0.61	19.66 (11.12, 28.87)^b^	0.25	0.24	0.31	0.75	23.63 (14.72, 33.23)^b^	3.96	0.51
+ Psychostimulants	0.03	0.13	0.22	0.58	22.58 (16.84, 28.60)^b^	0.01	0.02	0.05	0.15	30.79 (23.38, 38.65)^b^	8.21	0.07

Data source: Centers for Disease Control and Prevention [ 32, 35]

Abbreviation: *AAPC* Average annual percent change; *AAPC Black-White diff* Average annual percent change difference between Black and White populations

Note: Overall mortality from a specific drug is all deaths involving this drug whether alone or when combined with any other drug

^a^Death rates were calculated per 100,000 population and adjusted to the 2000 US standard population

^b^AAPC is statistically significant from zero (2-sided *P* < 0.05). Mortality trends were evaluated using the Joinpoint Regression Program (Version 4.8.0.1)

Heroin ranked second in terms of overdose death rates from an opioid drug reaching 5.23 and 4.77 per 100,000 among White and Black persons in 2018, respectively (Table 2). Heroin death rates accelerated starting in 2010 in both racial groups, with a faster increase observed among Black persons. The two drugs that resulted in the highest mortality when concomitantly used with heroin were synthetic opioids followed by cocaine (Fig. 2). Death rates due to prescription opioid overdose were higher in White compared to Black persons during the entire study period. Rates in White persons increased during wave 1 and then leveled off during subsequent waves; however, Black death rates from prescription opioids continued to modestly increase throughout all waves (Fig. 2). When records were limited to combinations of prescription and synthetic opioids, rates steeply increased in both racial groups during wave 3.

### AADR and mortality trends by race and sex

When stratified by race and sex (Additional File Figure 1, Additional File Tables 3 and 4), mortality trends across all investigated drugs were consistently higher in men than women of the same racial group. Trends in men mirrored, but were more pronounced than, corresponding trends among all persons. During wave 3, death rates involving any opioid overdose increased twice as fast in Black persons compared to White counterparts. Black men experienced the fastest death rate from an opioid at an average annual increase of 30% (95% CI = 25.00, 35.23) (Additional file: Table 3). The most remarkable AADRs in 2018 were observed for all cocaine-involved death among Black men (13.37 per 100,000), which was twofold higher than in White men, and synthetic opioid deaths in both White and Black men, which exceeded 15 per 100,000 (Additional file: Table 3). The magnitude and type of drug combinations with the highest death rate in 2018 differed by race and sex. White women were likely to die from combinations of opioids and benzodiazepines (2.70 per 100,000) (Additional file: Table 4). Cocaine-opioid mixing, however, resulted in the highest death rates among White men, Black men, and Black women (4.91, 7.99, and 2.90 per 100,000, respectively) (Additional file: Tables 3 and 4).

We developed an interactive web-based dashboard to compare mortality trends associated with different psychoactive drugs, both overall and when used in conjunction with other investigated substances of abuse. This tool allows users to explore mortality trends across race, sex, and the three waves of the opioid epidemic. Users can interactively access our study results and find additional information on overdose deaths by visiting the following link: https://rawy.shinyapps.io/drugmortapp/.

### Prevalence measures

**Fig. 3:**
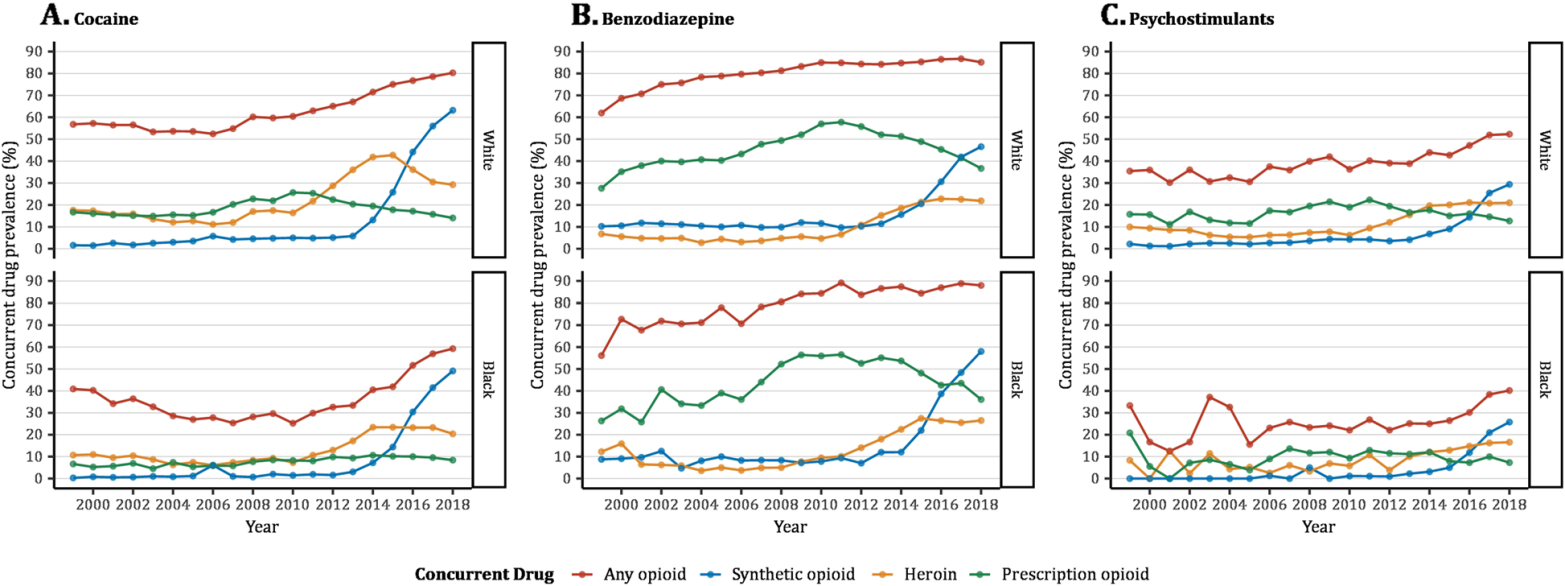
Percent of opioid co-involvement in all deaths due to (**A**) Cocaine, (**B**) Benzodiazepines, and (**C**) Psychostimulants by opioid subtype and race, 1999–2018. Note: Data are form the Centers for Disease Control and Prevention [32]

As shown in Table 1 and Fig. 3, approximately 80% of all cocaine-related deaths concomitantly involved an opioid drug in White persons compared to 60% in Black persons in 2018. However, the increase in opioid co-involvement between waves 1 and 3 was 33% among White persons compared to 135% among Black persons. In 2018, more than 50% of psychostimulant-related deaths in White persons included an opioid drug compared to 40% in Black persons. Opioid prevalence in psychostimulants deaths increased by 44% and 82% among White and Black persons, respectively compared to wave 1. Synthetic opioids exhibited a sharp co-involvement in cocaine and psychostimulants deaths since 2013 (Fig. 3). Unlike cocaine and psychostimulants, opioids were highly prevalent in benzodiazepine deaths (> 80%) during the entire study period, although the specific contributing opioid shifted from prescription to synthetic opioid between waves 1 and 3 (Fig. 3). Among individuals dying from a heroin overdose (Additional File Figure 2), synthetic opioid co-involvement increased steeply since 2013, followed by cocaine as the second most common contributor to heroin deaths in both groups. A rising pattern in synthetic opioid co-involvement in prescription opioid deaths was also observed at a time benzodiazepine co-involvement was plateauing (Additional file: Figure 2). No major differences in findings were observed when results were disaggregated by sex (see online interactive dashboard – https://rawy.shinyapps.io/drugmortapp/).

## Discussion

This study explored the evolving trends in mortality due to polysubstance use between 1999 and 2018, comparing them to mortality trends from specific drugs of abuse. We investigated a comprehensive list of opioid and nonopioid drugs that have received public health attention over the past two decades. Disaggregating results by race and sex provided insights on the differential impact of polysubstance mortality based on sociodemographic characteristics. Evidence of mortality due to simultaneous exposure to two or more drugs can be traced back to the beginning of the opioid epidemic. We found that combinations of opioid analgesics and benzodiazepines were increasingly a common cause of polysubstance death during wave 1, primarily impacting White persons. Beginning in wave 3, however, polysubstance mortality involving illicit drugs has had a staggering influence on accelerating overdose deaths among both White and Black Americans, particularly combinations of synthetic opioids with cocaine or heroin.

The pattern of polysubstance mortality observed during the first wave aligned with the overprescribing of opioid analgesics among White persons [20, 37], an associated increase in benzodiazepine prescriptions [38], and the common practice of their concomitant use [39]. This surge in overdose deaths subsequently triggered multiple mitigating measures, including the introduction of prescription drug monitoring programs and the Food and Drug Administration’s issuance of a boxed warning on the risks associated with benzodiazepine-opioid coadministration. Empirical evidence suggests that these measures might have contributed to suppressing the rising deaths from prescription opioids or their combined use with benzodiazepines by curtailing inappropriate prescribing [37, 40, 41]. Nonetheless, as the demand for opioids continued to rise and access to prescription-controlled substances became more restricted, effective strategies were lacking to counteract the growing availability and affordability of illicit drugs that substantially contributed to polysubstance mortality during waves 2 and 3.

Our results highlight the limitations of adopting a single-drug perspective in the US narrative of the drug crisis, which fails to capture the intertwined nature of the overdose epidemic and the role of important combinations in potentiating overdose risk. For instance, the potency of psychoactive drugs considerably increases when mixed or adulterated with the synthetic opioid fentanyl due to its extreme lethality. We found that the steepest rise in mortality occurred during wave 3 across all drugs when fentanyl was involved, consistent with evidence on increased mixing of fentanyl with other illicit drugs or its inclusion in counterfeit pharmaceutical pills [11, 42, 43], in addition to the unawareness of many drug users that they are taking drugs laced with fentanyl [43, 44]. Another fatal combination arises when a benzodiazepine is co-ingested with an opioid drug due to its synergistic effect on opioid-mediated respiratory and central nervous system depression. Opioids had the highest co-involvement in benzodiazepine deaths during the entire study period, underscoring the life-threatening effect of this combination and supporting previous findings that benzodiazepines are rarely fatal if not co-used with opioids [29]. The shift in fatal benzodiazepine-opioid combinations, from prescription opioids to synthetic opioids, is not surprising and reflects the transition occurring in the drug supply market. Our findings align with more comprehensive toxicological data from the CDC’s State Unintentional Drug Overdose Reporting System (SUDORS). Among the 25 states participating in SUDORS in 2018, most opioid overdose deaths (63%) involved a nonopioid drug, including benzodiazepines, cocaine, or methamphetamine [45].

The three-wave representation of the opioid epidemic provides a valuable framework for understanding the overarching trends in polysubstance mortality, particularly the shift from prescription to illicit drugs. However, this framework does not fully capture the nuanced and evolving nature of overdose crisis given the complex interaction between opioids and other substances of abuse. While opioids contributed to the majority of drug overdose deaths, we found a fast increase in the overall death rates of investigated nonopioid drugs since 2013, which mirrored mortality trends when these drugs were combined with opioids. These findings align with previous research highlighting the rapid increase in death rates from nonopioids, primarily driven by their concurrent use with opioids [29, 33, 46].

Our study identified an emerging racial disparity in mortality trends starting in 2013. Black persons were dying at average annual rates significantly faster than Whites for nearly all investigated drugs, including any opioid, psychostimulants, heroin, synthetic opioids, cocaine-opioid, psychostimulant-opioid, and psychostimulant-synthetic opioid combinations. These findings are consistent with growing evidence on the disparate increase in overdose death rates among Black persons during the third wave of the opioid epidemic [26, 47]. Multiple factors associated with underlying structural racism and social determinants of health could have contributed to these disparities [48, 49]. First, access to treatment was cited as a barrier in racially segregated Black communities due to low treatment capacity to meet patients’ needs [50], the disproportionate concentration of highly regulated and stigmatized methadone clinics compared to buprenorphine providers [51], and limited access to pharmacies where drug users or bystanders can purchase naloxone [52]. Additionally, long-standing mistrust of the healthcare system among Black Americans and the stigma associated with seeking OUD treatment [53] have been documented as significant barriers. Second, the high concurrent use of cocaine or methamphetamine with opioids, as observed among Black persons in our study, along with the low socioeconomic status in Black communities, are risk factors associated with poor treatment retention for addiction [14, 54]. Third, Black persons are more likely to face criminal charges for drug-related offenses compared to their White counterparts, which makes them less likely to initiate an emergency response [55, 56] and renders them more vulnerable to overdosing following release from correctional facilities [57].

Lastly, our analysis consistently revealed a higher overdose mortality rate among men than women across both racial groups, reflecting the reported higher illicit drug use among men [58]. Black men specifically experienced a high death burden from cocaine and opioids overall or when combined. This is likely attributed to the higher use of cocaine, especially crack, among Black persons compounded with the proliferation of opioids in the cocaine supply [58]. While mortality rates were generally lower for women, they experienced a significant increase in mortality trends from different drugs/combinations during the third wave. The highest polysubstance mortality among Black women resulted from cocaine-opioid use. Benzodiazepine-opioid combinations, however, continued to be the leading cause of polysubstance mortality among White women, which is in line with the reported high prescribing and use patterns of these drugs within the White female population [20, 39, 59]. Prevention and treatment approaches for substance use disorders should account for the unique needs and differences in addiction pathways and treatment outcomes between men and women. For example, women are at a higher risk of experiencing acute and chronic pain [60] and are more likely to be prescribed opioids than men [39]. Moreover, they encounter special barriers to OUD treatment, including higher perceived stigma, safety concerns, lower satisfaction with traditional treatment compared to women-only programs, and a higher prevalence of co-morbid psychiatric conditions [61].

The interpretation of our study results should consider several limitations. First, the Joinpoint Regression program does not allow controlling for covariates. Nonetheless, the AADR is a widely used and accepted measure for comparing mortality across populations over time while accounting for the differences in age distribution [36]. Second, the decision to focus exclusively on Black and White populations in our analysis was primarily driven by analytical and data constraints. Racial minorities with small population sizes have a small number of death counts when data are disaggregated at multiple levels, including year, race, sex, and specific drugs/drug combinations, rendering the computation of age-adjusted death rate statistically unreliable [62]. Moreover, unlike the high-quality reporting of race on death certificates for Black and White populations, evidence exists on the discrepancy in how the race/ethnicity of other groups, especially the American Indian or Alaska Native (AI/AN) population, was recorded by a funeral director on death certificates compared to self-reported census data [63]. This misclassification is known to impact the accuracy of death rate calculations, as demonstrated by a 40% underestimation in overdose deaths among AI/AN when race on Washington death certificates was corrected using tribal registry data [64]. Third, mortality data cannot differentiate between overdose deaths resulting from intentional mixing of two or more drugs, drug adulteration, or accidental contamination. Qualitative and mixed methods studies should corroborate surveillance data to better understand the nature of polysubstance use. Fourth, the drug(s) involved in deaths were not specified in 17% to 25% of drug overdose fatalities throughout the study period (ICD-10 code T50.9), which potentially underestimates death rates associated with specific drugs or drug combinations [65, 66]. Finally, improvements in post-mortem toxicological screening techniques over time, along with the geographic variations in the accuracy of screening, might have affected death rates and trends, especially in cases involving fentanyl analogs and other novel synthetic opioids [67, 68].

### Policy implications

In addition to the CDC’s advisory recommendations for a multidimensional approach targeting all stakeholders when responding to overdose crisis [69], our study highlights three critical issues that need immediate attention. First, polysubstance use should be considered the current norm when treating addiction. Opioid treatment programs should routinely screen for polysubstance use and adopt holistic treatment approaches, ideally combining pharmacologic and behavioral therapy [14]. Periodic surveillance using timely data is vital to effectively address the dynamic shifts in polysubstance use and death patterns across time, race, and geographic regions. The replacement of “polysubstance use” as a diagnosis with the broader term “substance use disorder” in the Diagnostic and Statistical Manual of Mental Disorders, Fifth Edition (DSM-V) fails to distinguish between single and multiple drug use, raising concerns about the need for clearer terminologies to describe the worsening phenomenon of polysubstance use [70, 71]. Second, addressing racial disparities in drug overdose requires interventions across the continuum of primary, secondary, and tertiary prevention. This involves addressing the underlying social determinants of health associated with addiction, expanding Medicaid coverage in all states, reforming criminal justice policies, facilitating safe community transition following incarceration, and developing culturally tailored interventions that ensure equitable access to medication-assisted therapy and harm reduction measures. Third, given how the synthetic opioid fentanyl and its analogs have plagued the illegal drug market [11], exacerbating polysubstance overdose deaths among Black and White communities, measures to address fentanyl-related use and overdose can change the landscape of the drug crisis. This includes expanding access to medication-assisted therapy, especially in racially segregated and underserved areas, and adopting novel harm-reduction approaches, like distributing fentanyl testing strips, expanding safe-drug consumption sites, and updating naloxone distribution policies [72, 73].

Our study contributes to the existing literature by providing a comprehensive assessment of the impact of drug combinations on overdose deaths among White and Black Americans during the two decades of the opioid crisis preceding the onset of the COVID-19 pandemic. The CDC-defined third wave of the opioid epidemic, which began in 2013 and was driven by synthetic opioids, marks a period when polysubstance mortality dominated drug overdose deaths. This period concurrently witnessed a disproportionate surge in mortality rates among Black Americans, especially Black men, surpassing those observed among their White counterparts. Understanding trends in polysubstance mortality and demographic variations by race and sex better describes the US opioid epidemic and drug crisis, supports data on the changes in the supply market of illicit drugs, and guides targeted policies and evidence-based strategies to address the drug crisis equitably among all impacted groups. The COVID-19 public health emergency has worsened the US drug crisis and substantially increased overdose deaths [2]. Future research should investigate the impact of the pandemic-related psychosocial stressors and disruptions to the OUD treatment, as well as the expansion of telehealth treatment modality and digital divide on pre-existing racial disparities in drug crisis [69, 74]. Additionally, more research is needed to investigate and address the unique challenges and health disparities encountered by other racial/ethnic groups, especially the AI/AN population, which experienced the largest increase in overdose death rates as indicated in recent reports [75, 76]. Correcting for AI/AN race misclassification on death certificates is recommended to avoid underestimating overdose deaths in this group [64].

## Conclusions

Polysubstance use has increasingly become the norm in drug overdose mortality, fueled by illicit drug combinations over the last decade. While the three-wave depiction of the opioid epidemic reflects the influence of opioids on overdose deaths, it does not fully characterize the changes in the extent and type of fatal polysubstance overdoses. The standard practice of reporting the overall mortality from a psychoactive drug masks the evolving nature of the drug crisis and hinders the effectiveness of measures to end this two-decade-long epidemic. Starting in 2013, synthetic opioids have substantially accelerated polysubstance mortality involving other opioid and non-opioid drugs in both racial groups. Compared to Whites, Black Americans are experiencing a faster surge in death rates from psychoactive drugs, spanning a range of substances, including any opioids, synthetic opioids, heroin, psychostimulants, and the concurrent use of cocaine/psychostimulants with opioids. White women are more vulnerable to overdosing when concurrently consuming opioids and benzodiazepines, a pattern that contrasts with the predominance of opioid-cocaine combinations among deaths in White men, as well as Black men and women. Characterizing these trends is essential for developing effective and targeted strategies to address the ongoing opioid epidemic and the broader drug crisis among different subpopulations.

### Supplementary Information

**Additional file.**

## Data Availability

The datasets analyzed during the current study are the 1999 through 2018 US Multiple Cause-of-Death Mortality Data from the National Vital Statistics System. They are publicly available through the Centers for Disease Control and Prevention (CDC) at https://www.cdc.gov/nchs/data_access/vitalstatsonline.htm#Mortality.

## References

[1] Wide-ranging online data for epidemiologic research (WONDER). Atlanta, GA: CDC, National Center for Health Statistics; 1999–2020. https://wonder.cdc.gov/controller/saved/D77/D305F620. Accessed 10 Sep 2022.

[2] Ahmad FB, Rossen LM, Sutton P. Provisional drug overdose death counts. National Center for Health Statistics. 2022. https://www.cdc.gov/nchs/nvss/vsrr/drug-overdose-data.htm. Accessed 20 Aug 2022.

[3] Wilson N, Kariisa M, Seth P, Smith H, Davis NL (2020). Drug and opioid-involved overdose deaths — United States, 2017–2018. MMWR Morb Mortal Wkly Rep.

[4] Centers for Disease Control and Prevention. Understanding the Epidemic. https://www.cdc.gov/opioids/basics/epidemic.html. Accessed 15 Apr 2021.

[5] Kariisa M, Scholl L, Wilson N, Seth P, Hoots B (2019). Drug overdose deaths involving cocaine and psychostimulants with abuse potential — United States, 2003–2017. MMWR Morb Mortal Wkly Rep.

[6] Ciccarone D (2021). The rise of illicit fentanyls, stimulants and the fourth wave of the opioid overdose crisis. Curr Opin Psychiatry.

[7] Jones CM, Bekheet F, Park JN, Alexander GC (2020). The evolving overdose epidemic: synthetic opioids and rising stimulant-related harms. Epidemiol Rev.

[8] Dowell D, Noonan RK, Houry D (2017). Underlying factors in drug overdose deaths. JAMA.

[9] Centers for Disease Control and Prevention. Drug overdose deaths. https://www.cdc.gov/drugoverdose/deaths/other-drugs.html. Accessed 20 Apr 2021.

[10] Cicero TJ, Ellis MS, Kasper ZA (2020). Polysubstance use: a broader understanding of substance use during the opioid crisis. Am J Public Health.

[11] Drug Enforcement Administration. 2019 National Drug Threat Assessment. https://www.dea.gov/documents/2020/01/30/2019-national-drug-threat-assessment. Accessed 17 Mar 2021.

[12] Jones CM, Einstein EB, Compton WM (2018). Changes in synthetic opioid involvement in drug overdose deaths in the United States, 2010–2016. JAMA.

[13] Suzuki J, El-Haddad S (2017). A review: fentanyl and non-pharmaceutical fentanyls. Drug Alcohol Depend.

[14] Blondino CT, Gormley MA, Taylor DDH, Lowery E, Clifford JS, Burkart B (2020). The Influence of co-occurring substance use on the effectiveness of opiate treatment programs according to intervention type. Epidemiol Rev.

[15] O’Brien P, Henke RM, Schaefer MB, Lin J, Creedon TB (2020). Utilization of treatment by Medicaid enrollees with opioid use disorder and co-occurring substance use disorders. Drug Alcohol Depend.

[16] Lin LA, Bohnert ASB, Blow FC, Gordon AJ, Ignacio RV, Kim HM (2021). Polysubstance use and association with opioid use disorder treatment in the US veterans health administration. Addiction.

[17] Chan B, Freeman M, Kondo K, Ayers C, Montgomery J, Paynter R (2019). Pharmacotherapy for methamphetamine/amphetamine use disorder—a systematic review and meta-analysis. Addiction.

[18] Chan B, Kondo K, Freeman M, Ayers C, Montgomery J, Kansagara D (2019). Pharmacotherapy for cocaine use disorder—a systematic review and meta-analysis. J Gen Intern Med.

[19] Fairbairn N, Coffin PO, Walley AY (2017). Naloxone for heroin, prescription opioid, and illicitly made fentanyl overdoses: challenges and innovations responding to a dynamic epidemic. Int J Drug Policy.

[20] Meghani SH, Byun E, Gallagher RM (2012). Time to take stock: a meta-analysis and systematic review of analgesic treatment disparities for pain in the United States. Pain Med.

[21] Pletcher MJ, Kertesz SG, Kohn MA, Gonzales R (2008). Trends in opioid prescribing by race/ethnicity for patients seeking care in US emergency departments. JAMA.

[22] James K, Jordan A (2018). The opioid crisis in black communities. J Law Med Ethics.

[23] Alexander MJ, Kiang MV, Barbieri M (2018). Trends in black and white opioid mortality in the United States, 1979–2015. Epidemiology.

[24] Substance Abuse and Mental Health Services Administration. The Opioid Crisis and the Black/African American Population: An Urgent Issue. 2020. https://store.samhsa.gov/product/The-Opioid-Crisis-and-the-Black-African-American-Population-An-Urgent-Issue/PEP20-05-02-001. Accessed 30 Mar 2021.

[25] Jordan A, Mathis M, Haeny A, Funaro M, Paltin D, Ransome Y (2021). An evaluation of opioid use in black communities: a rapid review of the literature. Harv Rev Psychiatry.

[26] Furr-Holden D, Milam AJ, Wang L, Sadler R (2021). African Americans now outpace whites in opioid-involved overdose deaths: a comparison of temporal trends from 1999 to 2018. Addiction.

[27] Kariisa M, Seth P, Scholl L, Wilson N, Davis NL (2021). Drug overdose deaths involving cocaine and psychostimulants with abuse potential among racial and ethnic groups – United States, 2004–2019. Drug Alcohol Depend.

[28] Johnson K, Jones C, Compton W, Baldwin G, Fan J, Mermin J (2018). Federal response to the opioid crisis. Curr HIV/AIDS Rep.

[29] Ruhm CJ (2019). Nonopioid overdose death rates rose almost as fast as those involving opioids, 1999–2016. Health Aff.

[30] von Elm E, Altman DG, Egger M, Pocock SJ, Gøtzsche PC, Vandenbroucke JP (2014). The strengthening the reporting of observational studies in epidemiology (STROBE) statement: guidelines for reporting observational studies. Int J Surg.

[31] Ingram DD, Malec DJ, Makuc DM, et al. National Center for Health Statistics Guidelines for Analysis of Trends. Vital Health Stat 2(179). 2018. https://www.cdc.gov/nchs/data/series/sr_02/sr02_179.pdf. Accessed 2 Mar 2020. 29775435

[32] Centers for Disease Control and Prevention. National Center for Health Statistics. Mortality multiple cause files. https://www.cdc.gov/nchs/data_access/vitalstatsonline.htm#Mortality_. Accessed 10 Mar 2020.

[33] Jones CMC, Baldwin GT, Compton WM (2017). Recent increases in cocaine-related overdose deaths and the role of opioids. Am J Public Health.

[34] National Cancer Institute (2020). Joinpoint Regression Program (Version 4.8.0.1) [computer software].

[35] Centers for Disease Control and Prevention. U.S. Census Populations With Bridged Race Categories 1999 to 2018 request. https://www.cdc.gov/nchs/nvss/bridged_race.htm. Accessed 13 Mar 2020.

[36] Curtin LR, Klein RJ. Direct standardization (age-adjusted death rates). Healthy People 2000 Stat Notes. 1995:1–10. 11762384

[37] Levy B, Paulozzi L, Mack KA, Jones CM (2015). Trends in opioid analgesic prescribing rates by specialty, U.S., 2007–2012. Am J Prev Med.

[38] Bachhuber MA, Hennessy S, Cunningham CO, Starrels JL (2016). Increasing benzodiazepine prescriptions and overdose mortality in the United States, 1996–2013. Am J Public Health.

[39] Hwang CS, Kang EM, Kornegay CJ, Staffa JA, Jones CM, McAninch JK (2016). Trends in the concomitant prescribing of opioids and benzodiazepines, 2002–2014. Am J Prev Med.

[40] Patrick SW, Fry CE, Jones TF, Buntin MB (2016). Implementation of prescription drug monitoring programs associated with reductions in opioid-related death rates. Health Aff.

[41] Zhang VS, Olfson M, King M (2019). Opioid and benzodiazepine coprescribing in the United States before and after US food and drug administration boxed warning. JAMA Psychiat.

[42] Green TC, Gilbert M (2016). Counterfeit medications and fentanyl. JAMA Intern Med.

[43] LaRue L, Twillman RK, Dawson E, Whitley P, Frasco MA, Huskey A (2019). Rate of fentanyl positivity among urine drug test results positive for cocaine or methamphetamine. JAMA Netw Open.

[44] Carroll JJ, Marshall BDL, Rich JD, Green TC (2017). Exposure to fentanyl-contaminated heroin and overdose risk among illicit opioid users in Rhode Island: a mixed methods study. Int J Drug Policy.

[45] Gladden RM, O’Donnell J, Mattson CL, Seth P (2019). Changes in opioid-involved overdose deaths by opioid type and presence of benzodiazepines, cocaine, and methamphetamine — 25 states, July–December 2017 to January–June 2018. MMWR Morb Mortal Wkly Rep.

[46] Hoots B, Vivolo-Kantor A, Seth P (2020). The rise in non-fatal and fatal overdoses involving stimulants with and without opioids in the United States. Addiction.

[47] Hoopsick RA, Homish GG, Leonard KE (2021). Differences in opioid overdose mortality rates among middle-aged adults by race/ethnicity and sex, 1999–2018. Public Health Rep.

[48] Phelan JC, Link BG (2015). Is racism a fundamental cause of inequalities in health?. Annu Rev Sociol.

[49] Andraka-Christou B (2021). Addressing racial and ethnic disparities in the use of medications for opioid use disorder. Health Aff.

[50] Cummings JR, Wen H, Ko M, Druss BG (2014). Race/ethnicity and geographic access to Medicaid substance use disorder treatment facilities in the United States. JAMA Psychiat.

[51] Goedel WC, Shapiro A, Cerdá M, Tsai JW, Hadland SE, Marshall BDL (2020). Association of racial/ethnic segregation with treatment capacity for opioid use disorder in counties in the United States. JAMA Netw Open.

[52] Guadamuz JS, Wilder JR, Mouslim MC, Zenk SN, Alexander GC, Qato DM (2021). Fewer pharmacies in black and hispanic/latino neighborhoods compared with white or diverse neighborhoods, 2007–15. Health Aff.

[53] Martha Hostetter and Sarah Klein, “Understanding and Ameliorating Medical Mistrust Among Black Americans,” Transforming Care. Commonwealth Fund. 2021. 10.26099/9grt-2b21.

[54] Saloner B, Cook BL (2013). Blacks and hispanics are less likely than whites to complete addiction treatment, largely due to socioeconomic factors. Health Aff.

[55] Latimore AD, Bergstein RS (2017). “Caught with a body” yet protected by law? Calling 911 for opioid overdose in the context of the Good Samaritan Law. Int J Drug Policy.

[56] Camplain R, Sabo S, Baldwin JA, Camplain C, Pro G, Trotter RT (2020). Racial/ethnic differences in drug- and alcohol-related arrest outcomes in a Southwest County from 2009 to 2018. Am J Public Health.

[57] Mital S, Wolff J, Carroll JJ (2020). The relationship between incarceration history and overdose in North America: a scoping review of the evidence. Drug Alcohol Depend.

[58] Center for Behavioral Health Statistics and Quality. Results from the 2019 National Survey on Drug Use and Health: Detailed Tables. Rockville, MD: Substance Abuse and Mental Health Services Administration. 2020. https://www.samhsa.gov/data/report/2019-nsduh-detailed-tables. Accessed 10 Aug 2021.

[59] Cicero TJ, Wong G, Tian Y, Lynskey M, Todorov A, Isenberg K (2009). Co-morbidity and utilization of medical services by pain patients receiving opioid medications: data from an insurance claims database. Pain.

[60] Fillingim RB, King CD, Ribeiro-Dasilva MC, Rahim-Williams B, Riley JL (2009). Sex, gender, and pain: a review of recent clinical and experimental findings. J Pain.

[61] McHugh RK, Votaw VR, Sugarman DE, Greenfield SF (2018). Sex and gender differences in substance use disorders. Clin Psychol Rev.

[62] Statistical methods: Suppression of rates and counts. Centers for Disease Control and Prevention. https://www.cdc.gov/cancer/uscs/technical_notes/stat_methods/suppression.htm. Accessed 7 Nov 2023.

[63] Arias E, Heron M, Hakes J (2016). The validity of race and hispanic-origin reporting on death certificates in the United States: an update. Vital Health Stat.

[64] Joshi S, Weiser T, Warren-Mears V (2018). Drug, opioid-involved, and heroin-involved overdose deaths among American Indians and Alaska Natives — Washington, 1999–2015. MMWR Morb Mortal Wkly Rep.

[65] Ruhm CJ (2018). Corrected US opioid-involved drug poisoning deaths and mortality rates, 1999–2015. Addiction.

[66] Buchanich JM, Balmert LC, Williams KE, Burke DS (2018). The effect of incomplete death certificates on estimates of unintentional opioid-related overdose deaths in the united states, 1999–2015. Public Health Rep.

[67] Clinton HA, Thangada S, Gill JR, Mirizzi A, Logan SB (2021). Improvements in toxicology testing to identify fentanyl analogs and other novel synthetic opioids in fatal drug overdoses, connecticut, January 2016–June 2019. Public Health Rep.

[68] Warner M, Paulozzi LJ, Nolte KB, Davis GG, Nelson LS (2013). State variation in certifying manner of death and drugs involved in drug intoxication deaths. Acad Forensic Pathol.

[69] Centers for Disease Control and Prevention. Increase in fatal drug Overdoses across the United States driven by synthetic opioids before and during the COVID-19 pandemic. CDC Health Alert Network. 2020. https://emergency.cdc.gov/han/2020/han00438.asp. Accessed 3 Jan 2021.

[70] American Psychiatric Association. Diagnostic and Statistical Manual of Mental Disorders, 5th Ed. (DSM-5®). Washington, DC: American Psychiatric Association; 2013.

[71] Peppin JF, Raffa RB, Schatman ME (2020). The polysubstance overdose-death crisis. J Pain Res.

[72] Peiper NC, Clarke SD, Vincent LB, Ciccarone D, Kral AH, Zibbell JE (2019). Fentanyl test strips as an opioid overdose prevention strategy: findings from a syringe services program in the Southeastern United States. Int J Drug Policy.

[73] Barry CL (2018). Fentanyl and the evolving opioid epidemic: what strategies should policy makers consider?. Psychiatr Serv.

[74] Joudrey PJ, Adams ZM, Bach P, Van BS, Chaiton JA, Ehrenfeld L (2021). Methadone access for opioid use disorder during the COVID-19 pandemic within the United States and Canada. JAMA Netw Open.

[75] Spencer MR, Miniño AM, Warner M. Drug overdose deaths in the United States, 2001–2021. NCHS Data Brief, no 457. Hyattsville, MD: National Center for Health Statistics. 2022. 10.15620/cdc:122556. 36598401

[76] Friedman JR, Hansen H (2022). Evaluation of increases in drug overdose mortality rates in the US by race and ethnicity before and during the COVID-19 pandemic. JAMA Psychiat.

