# Supplementary material for: Polysubstance mortality trends in White and Black Americans during the opioid epidemic, 1999–2018
Source: BMC Public Health. 2024 Jan 7;24:112. doi: 10.1186/s12889-023-17563-x (PMC10771660; doi:10.1186/s12889-023-17563-x)
Supplement: Supplementary file 1 — Additional file. [file 12889_2023_17563_MOESM1_ESM.docx]

**Additional File**

**Polysubstance mortality trends in White and Black Americans during the opioid epidemic, 1999-2018**

**Additional File Table 1.** Classification and definition of ICD-10 cause-of-death codes involving opioids, benzodiazepines, and stimulants

**Additional File Table 2.** Sensitivity analysis of mortality trends due to synthetic opioids overall and in combination with other psychoactive drugs by race, 1999-2018 (excluding 2006)

**Additional File Figure 1.** Mortality trends from cocaine, benzodiazepines, and psychostimulants overall and in combination with opioids by race and sex, 1999–2018

**Additional File Table 3.** Mortality trends due to combinations of opioids and other psychoactive drugs by race in men, 2013-2018

**Additional File Table 4.** Mortality trends due to combinations of opioids and other psychoactive drugs by race in women, 2013-2018

**Additional File Figure 2.** Percent of co-involvement of psychoactive drugs in deaths due to opioid subtypes by race, 1999-2018

**Additional File Table 1. Classification and definition of ICD-10 cause-of-death codes involving opioids, benzodiazepines, and stimulants^a^**

| Step 1: Death due to drug overdose | | Underlying cause of death^b^ | | |
| --- | --- | --- | --- | --- |
|  | | ICD-10 code | Definition | |
|  | | X40–X44 | Accidental poisoning by drugs (unintentional) | |
|  | | X60–X64 | Intentional self-poisoning by drugs (Suicide) | |
|  | | X85 | Assault by drug poisoning (homicide) | |
|  | | Y10–Y14 | Drug poisoning of undetermined intent | |
|  | | | | |
| Step 2: Specific drug involved in overdose death | | Multiple cause of death | | |
|  |  | ICD-10 code | Definition | |
| Opioids |  | | |  |
| Any Opioid | | T40.0 | Poisoning by opium | |
|  | | T40.1 | Poisoning by heroin | |
|  | | T40.2 | Poisoning by other opioids (includes natural opioids like morphine and semisynthetic opioids like oxycodone and hydrocodone) | |
|  | | T40.3 | Poisoning by methadone | |
|  | | T40.4 | Poisoning by synthetic opioids other than methadone (e.g., fentanyl, fentanyl analogs, and tramadol) | |
|  | | T40.6 | Poisoning by other and unspecified narcotics | |
| Commonly prescribed opioids | | T40.2 | Poisoning by other opioids (includes natural opioids like morphine and semisynthetic opioids like oxycodone and hydrocodone) | |
| Heroin | | T40.1 | Poisoning by heroin | |
| Synthetic opioids | | T40.4 | Poisoning by synthetic opioids other than methadone (e.g., fentanyl, fentanyl analogs, and tramadol) | |
| Benzodiazepines | | | | |
| Benzodiazepines | | T42.4 | Poisoning by benzodiazepines | |
| Stimulants | | | | |
| Cocaine | | T40.5 | Poisoning by cocaine | |
| Psychostimulants with abuse potential | | T43.6 | Poisoning by psychostimulants with abuse potential excluding cocaine (includes methamphetamine, MDMA, methylphenidate, amphetamine salts) | |
| Abbreviations: ICD-10: International Classification of Diseases, Tenth Revision; MDMA: 3,4-methylenedioxy-methamphetamine  ^a^ First, a drug overdose death was identified using any underlying cause-of-death code among those listed. Multiple cause-of-death codes were then used to indicate the specific drug causing overdose death. For the purpose of this study, a polysubstance death is a drug-related death record that includes multiple cause-of-death codes from at least 2 of the investigated drugs.  ^b^Underlying cause of death is a disease or injury which initiated the train of events leading directly to death, or the circumstances of the accident or violence which produced the fatal injury [1]. | | | | |

**Additional File Table 2. Sensitivity analysis of mortality trends due to synthetic opioids overall and in combination with other psychoactive drugs by race, 1999-2018 (excluding 2006)**

| Drug/drug combination | White | | | | | Black/African American | | | | | AAPC  Black-White diff | *p*-value |
| --- | --- | --- | --- | --- | --- | --- | --- | --- | --- | --- | --- | --- |
|  | Age-adjusted death rate^a^ | | | | AAPC (95%CI)  (2013-2018) | Age-adjusted death rate^a^ | | | | AAPC (95%CI)  (2013-2018) |  |  |
|  | 1999 | 2010 | 2013 | 2018 |  | 1999 | 2010 | 2013 | 2018 |  |  |  |
| Synthetic opioids | | | | | | | | | | | | |
| Overall^b^ | 0.29 | 1.14 | 1.13 | 10.89 | 61.73* (50.23,74.12) | 0.11 | 0.35 | 0.47 | 10.68 | 93.93* (77.51,111.88) | -32.20* | 0.00 |
| + Heroin | 0.01 | 0.02 | 0.08 | 3.10 | 89.98* (75.48,105.68) | 0.00 | 0.00 | 0.06 | 3.48 | 115.07* (92.08,140.81) | -25.08 | 0.09 |
| + Prescription  opioids | 0.05 | 0.31 | 0.33 | 1.65 | 43.04* (33.86,52.85) | 0.02 | 0.09 | 0.13 | 1.36 | 66.59* (40.45,97.59) | -23.55 | 0.12 |
| + Benzodiazepines | 0.05 | 0.29 | 0.30 | 1.90 | 49.34* (39.85, 59.49) | 0.02 | 0.05 | 0.08 | 0.87 | 72.69* (49.86, 99.01) | -23.35 | 0.08 |
| + Cocaine | 0.02 | 0.06 | 0.08 | 2.79 | 103.29* (65.48,149.74) | 0.01 | 0.04 | 0.10 | 4.39 | 128.55* (91.65, 172.55) | -25.26 | 0.39 |
| + Psychostimulants | 0.00 | 0.03 | 0.06 | 1.41 | 87.42* (74.38, 101.43) | 0.00 | 0.00 | 0.01 | 0.55 | 122.99* (101.05,147.33) | -35.57* | 0.00 |
| Data source: Centers for Disease Control and Prevention [2,3].  Abbreviation: AAPC, average annual percent change; AAPC Black-White diff, average annual percent change difference between Black and White  *AAPC is statistically significant from zero (2-sided *P*<0.05). Mortality trends were evaluated using the Joinpoint Regression Program (Version 4.8.0.1).  ^a^ Death rates were calculated per 100,000 population and adjusted to the 2000 US standard population.  ^b^ Overall mortality refers to all deaths involving synthetic opioids whether alone or in combination with any other drug. | | | | | | | | | | | | |

**Additional File Figure 1. Mortality trends from cocaine, benzodiazepines, and psychostimulants overall and in combination with opioids by race and sex, 1999–2018**


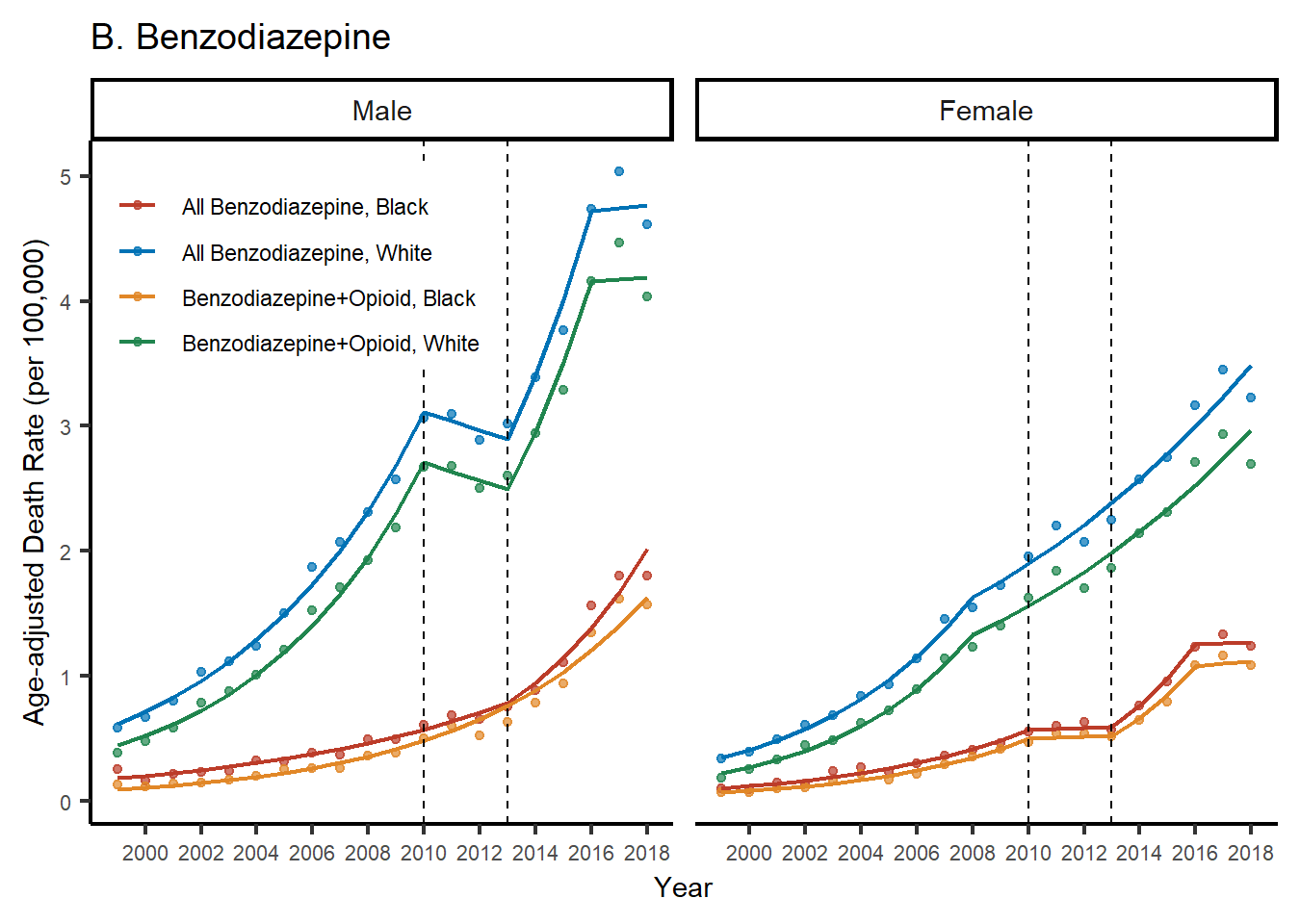

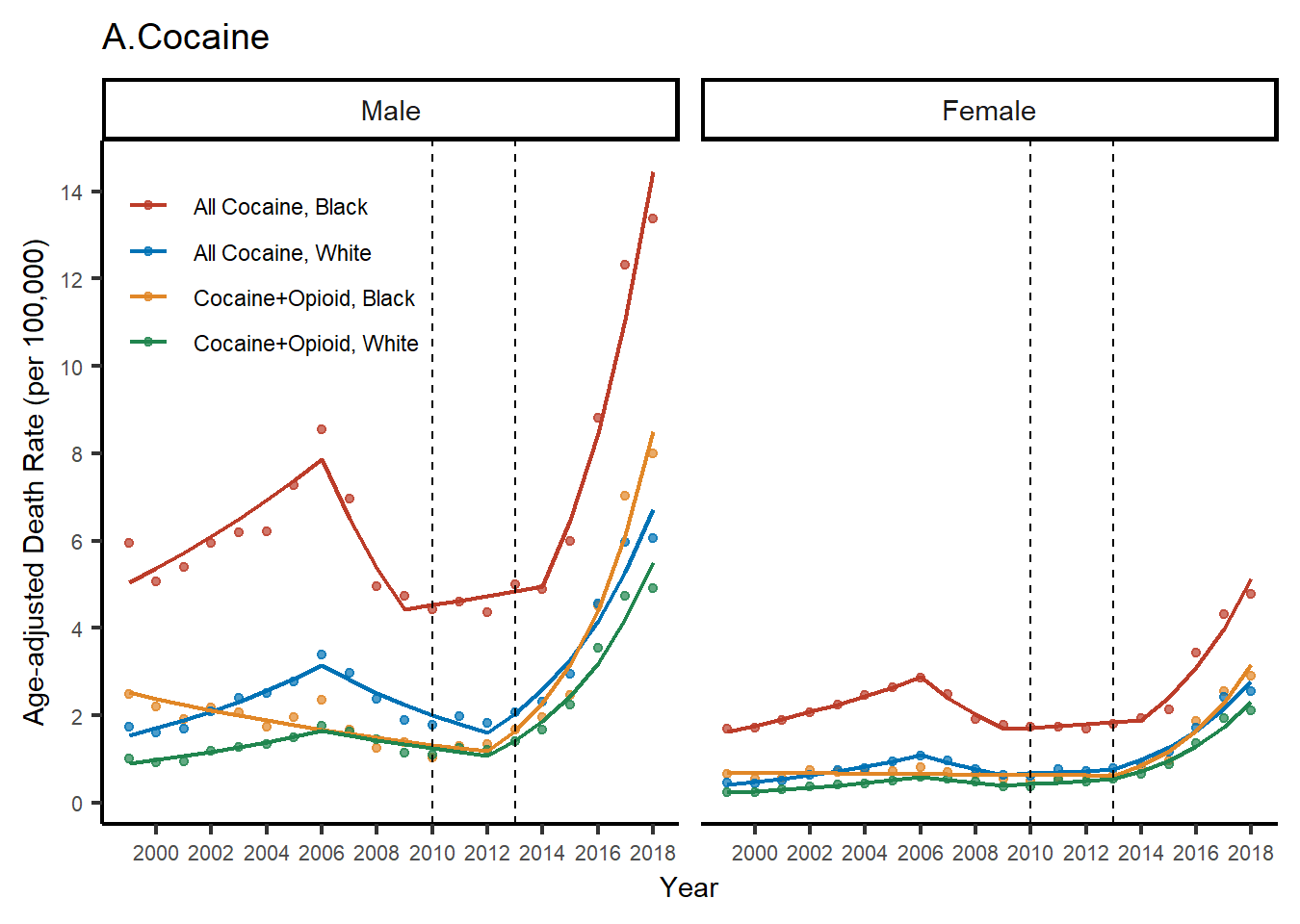


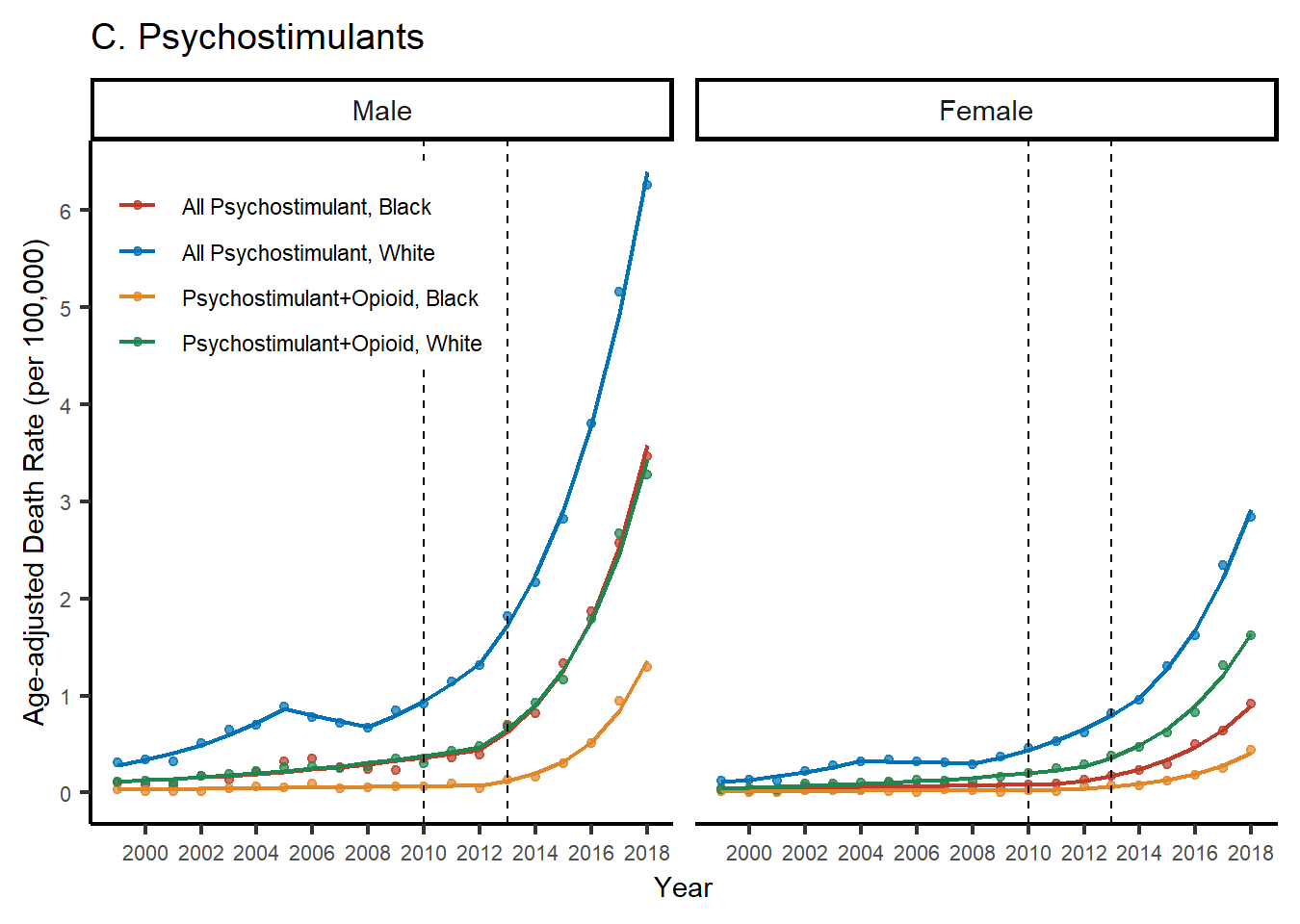


Data source: Centers for Disease Control and Prevention; Abbreviation: AAPC, average annual percent change

^ⴕ^ AAPC is **not statistically** significant from zero (2-sided *P*<0.05). Mortality trends were evaluated using the Joinpoint Regression Program (Version 4.8.0.1).

Note: Overall mortality from a specific drug is all deaths involving this drug whether alone or when combined with any other drug.

**Additional File Table 3. Mortality trends due to combinations of opioids and other psychoactive drugs by race in men, 2013-2018**

| **Cause of drug-related death** | **White Men** | | | | **Black Men** | | | |
| --- | --- | --- | --- | --- | --- | --- | --- | --- |
|  | **Age-adjusted Death Rate Per 100,000 Population** | | **AAPC** | **AAPC 95%** | **Age-adjusted Death Rate Per 100,000 Population** | | **AAPC** | **AAPC 95%** |
|  | **2013** | **2018** |  |  | **2013** | **2018** |  |  |
| All Opioids | 11.70 | 22.15 | 15.00 | 9.30 - 21.00 | 6.33 | 20.64 | 30.01 | 25.00 - 35.23 |
| Synthetic Opioids | 1.24 | 15.43 | 68.54 | 56.19 - 81.87 | 0.48 | 16.83 | 103.8 | 91.42 - 116.97 |
| Heroin | 4.73 | 7.80 | 12.04 | 9.29 - 14.86 | 3.45 | 7.64 | 20.86 | 13.05 - 29.2 |
| Psychostimulants | 1.82 | 6.26 | 29.88 | 26.87 - 32.97 | 0.70 | 3.47 | 41.55 | 34.68 - 48.76 |
| Cocaine | 2.06 | 6.05 | 26.85 | 20.36 - 33.69 | 4.99 | 13.37 | 24.46 | 17.79 - 31.51 |
| Prescription Opioids | 4.75 | 5.09 | 2.75^ⴕ^ | -4.21 - 10.21 | 1.77 | 3.35 | 14.92 | 11.42 - 18.54 |
| Cocaine + All Opioids | 1.40 | 4.91 | 31.06 | 24.63 - 37.82 | 1.67 | 7.99 | 38.95 | 30.51 - 47.94 |
| Synthetic Opioids + Heroin | 0.11 | 4.61 | 88.85 | 65.55 - 115.44 | 0.07 | 5.54 | 112.6 | 91.66 - 135.83 |
| Benzodiazepines | 3.02 | 4.61 | 10.48 | 4.09 - 17.27 | 0.75 | 1.80 | 20.87 | 14.79 - 27.27 |
| Benzodiazepines + All Opioids | 2.60 | 4.04 | 10.90 | 3.58 - 18.73 | 0.63 | 1.57 | 16.46 | 15.01 - 17.94 |
| Cocaine + Synthetic Opioids | 0.11 | 3.91 | 114.08 | 93.91 - 136.34 | 0.13 | 6.66 | 132.86 | 103.7 - 166.18 |
| Psychostimulants + All Opioids | 0.68 | 3.28 | 39.34 | 35.01 - 43.81 | 0.13 | 1.29 | 61.25 | 50.83 - 72.39 |
| Benzodiazepines + Synthetic Opioids | 0.31 | 2.42 | 50.87 | 33.41 - 70.61 | 0.09 | 1.16 | 85.57 | 59.39 - 116.05 |
| Prescription Opioids + Synthetic Opioids | 0.33 | 2.11 | 50.30 | 40.35 - 60.95 | 0.13 | 2.04 | 71.45 | 45.24 - 102.37 |
| Synthetic Opioids + Psychostimulants | 0.06 | 1.88 | 91.09 | 75.91 - 107.59 | 0.00 | 0.86 | 190.63 | 143.53 - 246.84 |
| Cocaine + Heroin | 0.80 | 1.86 | 19.43 | 14.84 - 24.2 | 0.96 | 2.73 | 28.70 | 23.55 - 34.07 |
| Benzodiazepines + Prescription Opioids | 1.55 | 1.51 | 0.69 ^ⴕ^ | -5.88 - 7.71 | 0.34 | 0.53 | 14.25 | 11.97 - 16.58 |
| Heroin + Psychostimulants | 0.30 | 1.41 | 37.28 | 32.52 - 42.21 | 0.07 | 0.55 | 50.08 | 42.71 - 57.83 |
| Benzodiazepines + Heroin | 0.61 | 1.25 | 17.71 | 11.9 - 23.82 | 0.17 | 0.53 | 22.27 | 12.42 - 32.99 |
| Cocaine + Benzodiazepines | 0.33 | 0.98 | 29.24 | 20.01 - 39.17 | 0.17 | 0.62 | 29.89 | 17.15 - 44.02 |
| Prescription Opioids + Heroin | 0.58 | 0.89 | 11.26 | 2.53 - 20.73 | 0.30 | 0.66 | 22.88 | 17.64 - 28.35 |
| Cocaine + Prescription Opioids | 0.41 | 0.80 | 18.24 | 9.29 - 27.92 | 0.44 | 1.08 | 22.33 | 15.74 - 29.29 |
| Prescription Opioids + Psychostimulants | 0.28 | 0.73 | 22.49 | 17.73 - 27.44 | 0.05 | 0.18 | 33.90 | 19.99 - 49.42 |
| Cocaine + Psychostimulants | 0.11 | 0.68 | 47.58 | 38.61 - 57.13 | 0.13 | 0.71 | 49.35 | 38.29 - 61.29 |
| Benzodiazepines + Psychostimulants | 0.19 | 0.68 | 30.44 | 25.68 - 35.39 | 0.03 | 0.10 | 40.63 | 21.95 - 62.17 |

Data source: Centers for Disease Control and Prevention [2,3]; Abbreviation: AAPC, average annual percent change

^ⴕ^ AAPC is **not statistically** significant from zero (2-sided *P*>0.05). Mortality trends were evaluated using the Joinpoint Regression Program (Version 4.8.0.1).

Note: Overall mortality from a specific drug is all deaths involving this drug whether alone or when combined with any other drug.

**Additional File Table 4. Mortality trends due to combinations of opioids and other psychoactive drugs by race in women, 2013-2018**

| **Cause of drug-related death** | **White Women** | | | | **Black Women** | | | |
| --- | --- | --- | --- | --- | --- | --- | --- | --- |
|  | **Age-adjusted Death Rate Per 100,000 Population** | | **AAPC**  **(2013-2018)** | **AAPC 95%** | **Age-adjusted Death Rate Per 100,000 Population** | | **AAPC**  **(2013-2018)** | **AAPC 95%** |
|  | **2013** | **2018** |  |  | **2013** | **2018** |  |  |
| All Opioids | 6.54 | 10.29 | 10.32 | 3.26 - 17.85 | 2.97 | 7.37 | 21.27 | 16.8 - 25.92 |
| Synthetic Opioids | 1.01 | 6.23 | 48.59 | 36.05 - 62.28 | 0.46 | 5.29 | 68.81 | 31.55 - 116.62 |
| Prescription Opioids | 3.50 | 3.55 | 0.15 ^ⴕ^ | -3.87 - 4.33 | 1.47 | 2.00 | 5.28 | 1.33 - 9.38 |
| Benzodiazepines | 2.25 | 3.23 | 7.90 | 6.71 - 9.1 | 0.57 | 1.24 | 16.4 | 3.05 - 31.48 |
| Psychostimulants | 0.82 | 2.84 | 29.52 | 25.38 - 33.8 | 0.17 | 0.92 | 38.73 | 32.25 - 45.53 |
| Benzodiazepines + All Opioids | 1.86 | 2.70 | 8.35 | 6.93 - 9.79 | 0.52 | 1.08 | 16.62 | 3.92 - 30.88 |
| Heroin | 1.32 | 2.60 | 16.73 | 12.21 - 21.44 | 0.70 | 2.27 | 25.53 | 19.93 - 31.39 |
| Cocaine | 0.79 | 2.56 | 29.55 | 22.23 - 37.31 | 1.80 | 4.77 | 22.71 | 16.97 - 28.72 |
| Cocaine + All Opioids | 0.55 | 2.11 | 33.75 | 25.8 - 42.2 | 0.63 | 2.90 | 38.58 | 28.96 - 48.91 |
| Cocaine + Synthetic Opioids | 0.06 | 1.65 | 105.57 | 75.05 - 141.41 | 0.08 | 2.40 | 105.60 | 83.07 - 130.89 |
| Psychostimulants + All Opioids | 0.38 | 1.62 | 35.23 | 30.09 - 40.57 | 0.07 | 0.44 | 46.07 | 37.48 - 55.19 |
| Synthetic Opioids + Heroin | 0.04 | 1.56 | 86.74 | 68.64 - 106.78 | 0.04 | 1.69 | 116.43 | 97.36 - 137.35 |
| Benzodiazepines + Synthetic Opioids | 0.29 | 1.36 | 40.25 | 31.09 - 50.05 | 0.07 | 0.61 | 55.05 | 39.11 - 72.81 |
| Benzodiazepines + Prescription Opioids | 1.18 | 1.29 | 3.15 | 0.68 - 5.68 | 0.38 | 0.55 | 9.10 | 5.53 - 12.79 |
| Prescription Opioids + Synthetic Opioids | 0.32 | 1.17 | 32.84 | 22.19 - 44.42 | 0.12 | 0.78 | 48.25 | 7.44 - 104.58 |
| Synthetic Opioids + Psychostimulants | 0.05 | 0.93 | 86.46 | 75.12 - 98.52 | 0.01 | 0.27 | 82.94 | 65.64 - 102.06 |
| Cocaine + Heroin | 0.26 | 0.69 | 23.18 | 16.14 - 30.64 | 0.23 | 0.98 | 31.59 | 26.21 - 37.2 |
| Heroin + Psychostimulants | 0.12 | 0.55 | 34.91 | 28.92 - 41.18 | 0.01 | 0.16 | 54.23 | 46.44 - 62.44 |
| Benzodiazepines + Heroin | 0.23 | 0.53 | 21.74 | 13.64 - 30.42 | 0.07 | 0.28 | 33.34 | 26.82 - 40.19 |
| Cocaine + Benzodiazepines | 0.16 | 0.50 | 28.7 | 20.96 - 36.93 | 0.10 | 0.32 | 14.84 | 11.71 - 18.06 |
| Prescription Opioids + Psychostimulants | 0.17 | 0.43 | 18.33 | 16.78 - 19.89 | 0.04 | 0.13 | 18.87 | 13.07 - 24.96 |
| Cocaine + Prescription Opioids | 0.18 | 0.43 | 21.00 | 11.68 - 31.09 | 0.20 | 0.46 | 19.65 | 7.02 - 33.77 |
| Benzodiazepines + Psychostimulants | 0.12 | 0.43 | 29.61 | 23.77 - 35.73 | 0.02 | 0.10 | 44.74 | 32.56 - 58.05 |
| Cocaine + Psychostimulants | 0.05 | 0.35 | 54.72 | 41.86 - 68.74 | 0.04 | 0.24 | 40.76 | 27.01 - 55.99 |
| Prescription Opioids + Heroin | 0.17 | 0.32 | 15.37 | 8.47 - 22.71 | 0.08 | 0.27 | 31.71 | 24.69 - 39.13 |

Data source: Centers for Disease Control and Prevention [2,3]; Abbreviation: AAPC, average annual percent change

^ⴕ^ AAPC is **not statistically** significant from zero (2-sided *P*>0.05). Mortality trends were evaluated using the Joinpoint Regression Program (Version 4.8.0.1).

Note: Overall mortality from a specific drug is all deaths involving this drug whether alone or when combined with any other drug.

**Additional File Figure 2. Percent of co-involvement of psychoactive drugs in deaths due to opioid subtypes by race, 1999-2018**


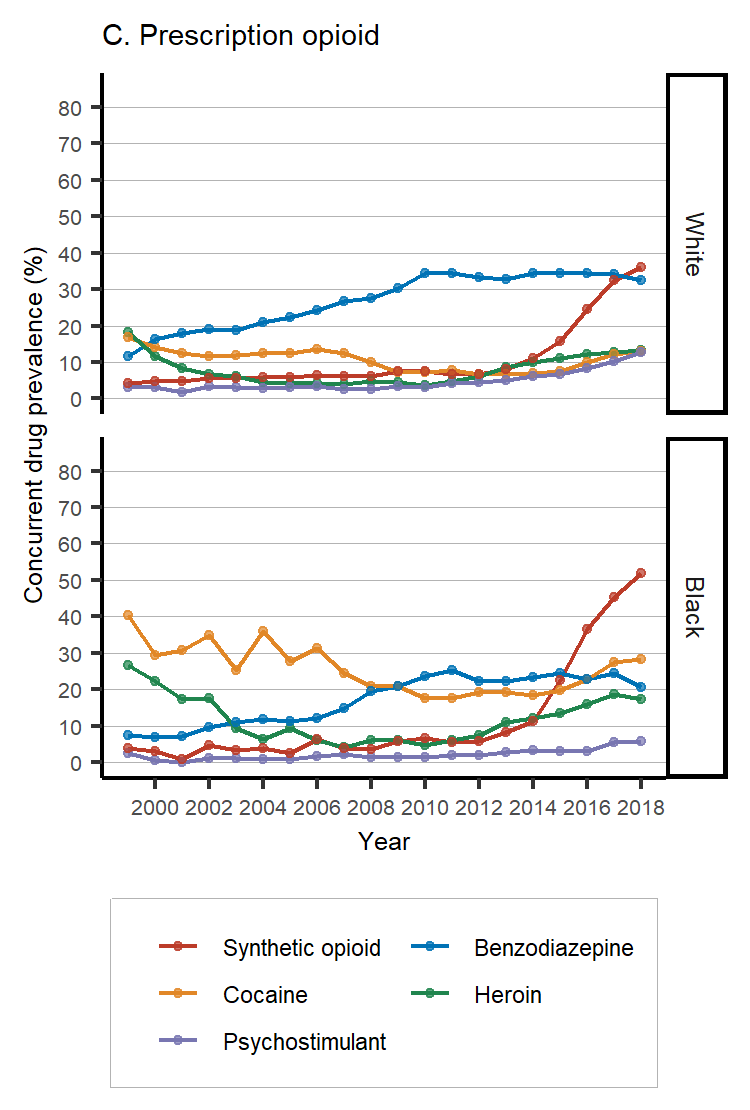

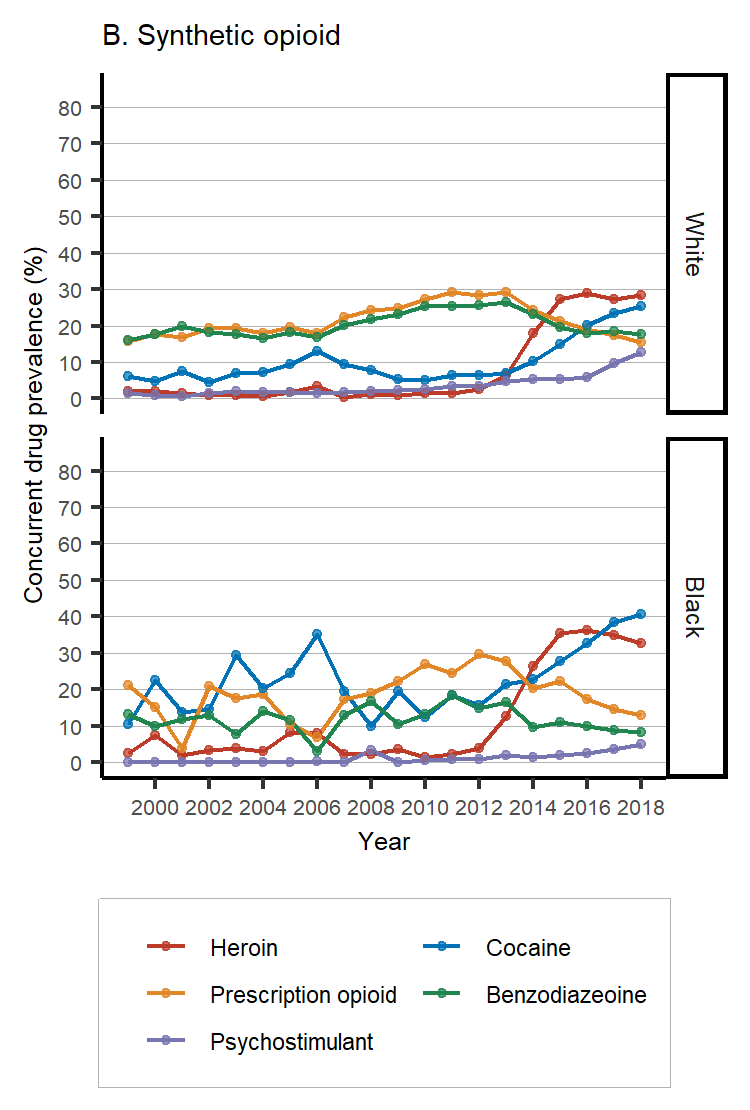

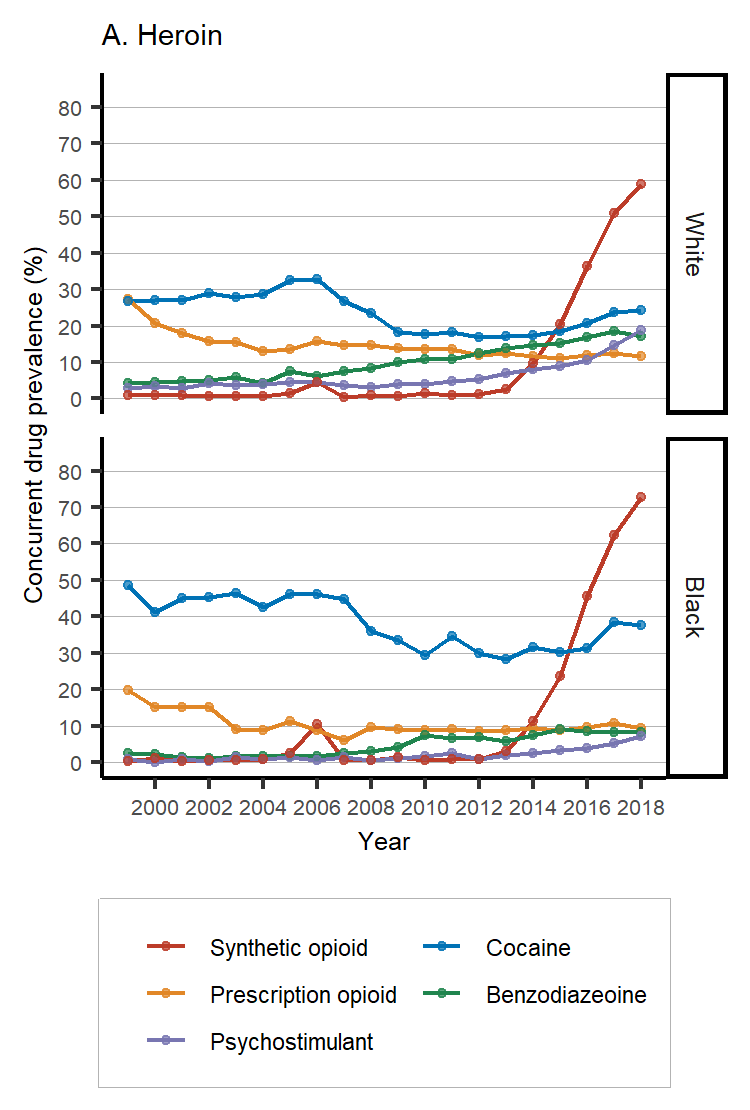


References

World Health Organization. International Statistical Classification of Diseases and Related Health Problems-10th Revision (5 ed.).2016. Geneva, Switzerland: WHO Press.

Centers for Disease Control and Prevention. Mortality multiple cause files. Accessed March 10, 2020. <https://www.cdc.gov/nchs/data_access/vitalstatsonline.htm#Mortality>

Centers for Disease Control and Prevention. U.S. Census populations with bridged race categories 1999 to 2018 request. Accessed March 13, 2020. <https://www.cdc.gov/nchs/nvss/bridged_race.htm>
